# Supplementary material for: Are carbon emissions trading and green financial instruments synergistic? -Comprehensive quantitative research based on content analysis
Source: PLoS One. 2024 Mar 7;19(3):e0298601. doi: 10.1371/journal.pone.0298601 (PMC10919601; doi:10.1371/journal.pone.0298601)
Supplement: S1 Data — (ZIP) [file pone.0298601.s001.zip › Data/地级市Z_F工作报告.zip!/╕╩╦α/╢¿╬≈╩╨/└┤╫╘╣1⁄2╓┌║┼í╛┬φ┐╦╩2╛▌═°í┐_2012.docx]

各位代表：

　　现在，我代表市人民政府，向大会作工作报告，请予审议,并请各位政协委员和列席会议的同志提出意见。

　　过去五年工作回顾

　　市二届人大一次会议以来的五年，是定西历史上发展又好又快的五年。五年来，在省委、省政府和市委的坚强领导下，在市人大、市政协的监督支持下，市政府深入贯彻落实科学发展观，抢抓机遇, 开拓进取，攻坚克难，全面完成了任期目标任务。

　　——综合实力在加快发展中显著提升。坚持以项目建设为抓手，努力扩大投资规模，拉动经济快速增长。预计2011年底，全市完成生产总值186亿元，比2006年翻一番多，年均增长10.61%。固定资产投资281.5亿元，比2006年增长5.6倍，年均增长45.73%。规模以上工业增加值16.2亿元，比2006年增长1.6倍，年均增长15.5%。社会消费品零售总额62.7亿元，比2006年增长1.2倍，年均增长17.07%。大口径财政收入、一般预算收入、财政支出分别完成17亿元、9亿元、110亿元，比2006年净增12.07亿元、6.3亿元、81.9亿元。金融机构存款余额346.92亿元、贷款余额186.68亿元，年均分别增长28.71%、24.36%。招商引资累计到位资金251.4亿元，年均增长35.98%。

　　——经济结构在调整转型中不断优化。坚持兴农强工，不断调整经济结构，促进产业优化升级。大力发展旱作高效农业，预计2011年粮食总产量120.17万吨，比2006年增加35.06万吨。围绕打造“中国薯都”、“中国药都”， 全力推进省级马铃薯、中医药循环经济产业园建设，连年举办全国性两大产业发展大会，产业知名度和影响力有效提升，富民强市作用日益显现。全面落实工业发展“十大行动计划”，实施工业项目347个，完成投资108亿元，以有色冶金、农畜产品加工、现代制药、矿产建材为主的工业体系初步形成，工业占生产总值比重由2006年的11.6%提高到18.2%、对财政贡献率由14.4%提高到21%。第三产业快速发展，增加值达80.72亿元，年均增长12.29%。坚持内抓培育、外抓引进，个体私营企业迅速壮大，非公有制经济占生产总值比重达38%。对外贸易不断扩大，累计实现进出口总额5036万美元。积极发展循环经济，节能减排任务全面完成。三次产业结构由2006年的40︰19︰41调整为28︰29︰43。

　　——城乡面貌在夯实基础中明显变化。坚持以做大城镇统筹城乡发展，着力建设“五宜”城市，努力改善生产生活条件。实施城建项目745个，市区旧城改造稳步推进、新城框架初步形成，各县县城和小城镇建设步伐加快，城镇建成区面积由62平方公里扩大到81平方公里，城镇化率提高到24.42%。实施交通项目1825个，完成投资81.81亿元，公路网密度由每百平方公里17.23公里提高到49.6公里，等级公路覆盖全部乡镇，平定、天定高速公路建成通车，兰渝铁路过境段和定临公路内官至临洮段、陇西文峰至漳县殪虎桥二级公路建设进展顺利。实施节水灌溉、中小河流治理和病险水库除险加固等水利工程210项，完成投资17.4亿元，基本解决了119万农村人口的饮水安全问题，引洮供水一期及配套工程加快推进、二期可研已经省上初审。生态建设不断加强，实施林业重点工程77.4万亩，新修梯田108.4万亩，水土流失综合治理程度41.4 %，森林覆盖率12.5%，新建农村户用沼气9.58万户。实施易地扶贫搬迁项目95个、整村推进项目284个，2个国家级和7个省级扶贫连片开发试点项目深入推进。新农村建设步伐加快，8个省级试点村完成建设任务，1个省级试点县和6个市级试点镇建设进展顺利。电力、通信等设施进一步完善，城乡居民生产生活条件明显改善。

　　——社会事业在统筹发展中全面进步。努力提高公共服务水平，大力发展社会各项事业。稳步推进教育布局调整，努力改善办学条件，实施各类教育项目1200多个，总投资14亿元，新建校舍100万平方米，D级危房全部消除。狠抓教育教学质量，高考二本上线率由2006年的15.95%提高到23.4%，职业技术教育发展走在了全省前列。实施科技项目304项，申报国家专利117件，科技创新和成果转化应用能力不断增强。积极整合医疗卫生资源，市医院迁建项目顺利实施，搬迁新建县级医院7个，改扩建乡镇卫生院100个，建成标准化村卫生室428个，公共卫生服务条件显著改善。文化事业不断繁荣，市图书馆、博物馆、文化馆、城市展览馆和剧院开工建设，建成乡镇综合文化站105个、农家书屋1503个，洮岷花儿等非物质文化遗产得到有效保护与传承，成功注册“李氏文化”、“貂蝉故里”商标，大型现代秦剧《百合花开》荣获全国第十一届精神文明建设“五个一工程”奖和甘肃戏剧艺术最高奖——“红梅奖”。完成有线电视数字化整体转换6.2万户，广播电视综合覆盖率达90%以上，定西电视台新闻综合频道节目在兰州地区入网播出。人口和计划生育事业持续健康发展，创建省级和国家级优质服务县工作进展顺利，人口自然增长率控制在6.7‰以内。体育活动场所建设不断加强，全民健身活动蓬勃开展。气象、统计、质量技术监督、食品药品监管、防震减灾、外事侨务、民族宗教、社会慈善、妇女儿童、老年人、青少年、残疾人等事业都有了新发展。

　　——人民生活在共建共享中持续改善。大力整合各类项目资金，充分调动一切积极因素，全力保障和改善民生。财政用于民生方面的支出达115亿元，是前五年的7.4倍。累计为民办实事73件，城乡居民就业、医疗、住房、出行、饮水等方面的突出困难逐步缓解。发放小额担保贷款8.19亿元，新增城镇就业6.36万人，下岗失业人员再就业2.88万人，安置高校毕业生1.87万人，输转剩余劳动力293.75万人次，实现劳务收入209.45亿元。城镇职工养老、失业、医疗、工伤、生育保险制度和新型农村合作医疗、城镇居民医疗保险制度不断完善，城乡居民社会养老保险试点进展顺利。发放城乡低保金14.85亿元，城乡低保标准逐年提高，城市低保实现应保尽保，农村低保覆盖面由0.24%提高到19.3%。建成1个市级和6个县级社会福利中心、60个农村敬老院和五保家园，五保对象集中供养率达到10%。在全省率先建立对城市低保户和农村五保户春节前普惠制慰问制度，适时增加社区工作人员、代课教师、环卫工人、聘任制文化专干等低收入群体生活补贴。建设廉租房1.29万套，实施棚户区改造1.5万户，改造农村危旧房5.98万户。城乡居民收入持续增加，预计2011年城镇居民人均可支配收入、农民人均纯收入分别为12085元、3050元，年均增长10.6%、11.6%。

　　——发展活力在改革创新中逐步增强。积极推进重点领域改革创新，消除体制机制障碍，优化经济社会发展环境。深化财政体制改革，全面推行国库集中支付、部门预算、非税收入管理和乡财县管、村财乡管制度，财政管理规范化、精细化水平不断提高。深化投融资体制改革，推动银政银企合作，引进设立兰州银行定西分行，引导商业银行增加信贷投放114亿元。加快推进国有企业改革，国营敬东机器厂政策性破产全面完成。集体林权制度完成主体改革任务，配套改革稳步推进。积极探索农村土地经营权改革，流转土地74.39万亩。医药卫生体制改革深入实施，基本药物制度初步建立，门诊医疗报销试点工作在漳县正式启动。加强和创新社会管理，注重基层管理服务，健全应急管理体系，强化安全生产责任，妥善解决信访突出问题，依法打击各类犯罪活动，扎实推进平安定西建设，社会保持和谐稳定。2009年，我市被评为“全国社会治安综合治理优秀市”。

　　——政府建设在转变职能中深入推进。新一轮政府机构改革全面完成，运转功能进一步增强。加快行政审批制度改革，取消、下放、调整行政审批事项71项。深化干部人事制度改革，事业单位岗位设置和行政事业单位工勤人员聘用制管理全面实施。有针对性地举办项目管理、工业发展、城镇建设等培训研讨班，科学决策和推动发展能力进一步提高。围绕规范政府行政行为，不断加强普法教育、法制建设和制度建设，制定修订《市政府工作规则》等制度27项，依法行政和规范运行水平明显提高。加快推行政务公开，畅通政府联系群众渠道，妥善解决群众反映的热点难点问题。严格执行党风廉政建设责任制，深入开展政风行风建设和民主评议工作，强化行政监察和审计监督，廉政建设取得新成效。自觉接受人大、政协监督，办理人大代表建议451件、政协委员提案857件。

　　各位代表，2011年是本届政府任期的最后一年。一年来，我们埋头苦干，狠抓落实，经济社会发展实现了“十二五”良好开局。

　　一是发展方式加快转变。按照“兴农强工、做大城镇、扩充总量、提升水平”的总体部署，着力推动经济工作从偏重传统农业向大力发展工业和现代农业转变，从偏重农村经济发展向城乡统筹发展转变，从偏重投资拉动向投资和消费拉动并重转变，从偏重经济发展向经济社会协调发展转变。依靠科技推广、循环利用、项目建设和政策扶持，促进经济总量扩充、质量提升、结构优化。第一产业以发展现代农业为主攻方向，在稳定粮食生产的基础上，坚持标准化种植、规模化养殖、精深化加工、市场化经营，着力推动特色优势产业提质增效。马铃薯产业实现脱毒种薯应用全覆盖，精深加工开始向高端迈进，“中国薯都”建设目标更加明确。道地中药材开发水平明显提升，种植加工衔接更加紧密，GAP标准化种植占到40%以上，“中国药都”建设加快推进。第二产业立足资源优势的有效转化，依托开发区和工业集中区呈规模化、集群化、循环化发展，节能减排预警监控有力跟进，清洁生产得到进一步重视和加强。第三产业突破传统发展方式，开始向现代物流引领、市场体系支撑、旅游产业带动、新兴业态补充的格局迈进。

　　二是项目建设强力推进。进一步加大与国家部委和省直厅局汇报衔接力度，一大批重点项目得到国家和省上支持。争取国家和省上预算内资金53亿元，增长20 %，实施500万元以上项目874个，其中新开工亿元以上项目83个。67个市列领导责任制项目完成投资66.8亿元。修订完善固定资产投资、开发区建设、招商引资考核评价、项目前期费使用等管理办法，制定重点项目建设领导责任、联席会议、现场办公、督查通报等4项制度。积极组织参加兰洽会、西洽会等大型投资节会，成功引进总投资42亿元的华能公司通渭华家岭风电等重大项目48个，招商引资实际到位资金85亿元，增长35%。

　　三是特色农业稳步发展。全市种植马铃薯324万亩，生产脱毒原原种3.8亿粒，建立加工企业原料供应标准化生产基地29万亩、优质商品薯标准化生产基地129万亩。种植中药材109万亩，建成标准化基地45万亩。继续加大种植业结构调整力度，推广全膜双垄沟播272万亩，其中黑膜覆盖马铃薯43万亩，为抗旱保粮奠定了坚实基础。设施农牧业快速发展，建成日光温室1178座、塑料大棚8855亩，新增规模养殖企业60个、养殖小区80个，发展规模养殖8010户，完成玉米秸秆青贮氨化130万吨。培训农民工 20.79万人，输转城乡劳动力65.25万人次，实现劳务收入60.6亿元。

　　四是工业经济快速增长。召开第二次工业发展大会，制定出台加快推进工业跨越发展的意见。进一步理顺园区管理和投融资体制，财政筹资2.7亿元，实施园区基础设施项目45项，4个省级开发区和3个工业集中区承载功能不断完善，新引进入驻企业40户。实施1000万元以上工业项目73个，完成投资48.23亿元，宏鑫公司再生塑料等38个项目建成投产，顾地公司管材等35个项目完成年度计划，奇正公司中药生产等25个项目开工建设。完成工业增加值33.9亿元，增长21.6%，增速同比加快7.6个百分点。

　　五是第三产业不断壮大。不断提升旅游产业发展层次，实现旅游收入10.32亿元。大力发展现代物流业，引进总投资15亿元的康美药业公司50万吨中药材仓储现代物流园及加工等项目。积极发展金融服务业，引导金融机构投放重点项目、循环经济园区和新城区建设贷款29亿元，比上年增长18.71％。加强对餐饮等民生服务行业的指导和监管，推动企业服务优质化、品牌化。健全完善市场体系，商品流通步伐加快，各类服务业发展日趋活跃。

　　六是城乡建设步伐加快。实施城建项目260个，完成投资70亿元，其中市区实施项目96个、完成投资37亿元。新城区劲东路等10条道路和定西湖、生态园、翻板坝、关川河景观带等项目基本建成，集中供热、大小碱沟综合治理、薯都大道等项目开工建设。旧城区污水处理改扩建、立交桥广场改造等项目全面建成，南大街等“城中村”改造工程顺利推进。县城和重点小城镇实施建设项目164个，完成投资33亿元。严格落实各项调控措施，房地产市场平稳健康发展。实施水利工程53项，引洮供水一期及配套工程完成投资6亿元。实施交通项目95项，新增公路里程315公里。实施易地搬迁项目25个、整村推进项目56个。新修梯田29.69万亩，新增水土流失综合治理面积350平方公里，平定、天定高速公路生态长廊建设年度任务全面完成。

　　七是民计民生有效保障。实施中小学校舍安全工程69个，建成特殊教育学校5所，高考二本上线8505人。建成农家书屋300个、乡镇综合文化站53个。新建乡镇卫生院5所、村卫生室255所。发放小额担保贷款3.53亿元，支持创业1万人，带动就业2万人。城市低保补助标准提高10%，农村低保补助标准提高到1096元。新建廉租住房7557套、经济适用房1025套、限价商品房608套、公共租赁房1132套，完成棚户区改造4109套、农村危旧房改造1.8万户。改造市区供热管网12处，硬化背街小巷10条。加强食品药品监管，创建食品安全示范店105个、农资放心店10个，建成市级食品安全快速检测中心1个、县级检测室7个。不断加强和创新社会管理，办理各类法律援助案件363件，在40个乡镇安装了视频监控系统。为民承诺办理的10件24项实事到年底能够全面完成任务。

　　各位代表！定西五年来的巨大变化，体现了省委、省政府和市委的正确决策，书写了历届市领导班子、全市广大干部群众的奋斗历程，凝聚了各位人大代表、政协委员的聪明才智，倾注了驻定单位、社会各界的大力支持。在此，我代表市人民政府，向全市各行各业的建设者，向各民主党派、工商联、无党派人士、人民团体、离退休老同志，向驻定单位，向人民解放军和武警部队官兵，向所有关心支持定西发展的各界人士，表示衷心的感谢！

　　回顾过去五年的发展历程，我们深刻体会到，要推动定西跨越发展，必须把科学发展作为第一要务，坚持走工业引领、产业提升、项目带动、城镇支撑的发展路子，调整经济结构，转变发展方式，提高发展质量。要增进群众福祉，必须把为民谋利作为第一追求，真心实意听民声、察民情、顺民意，竭尽全力干实事、解难事、办好事，使发展成果最大限度惠及人民群众。要构建和谐社会，必须把群众满意作为第一标准，努力加强和创新社会管理，夯实工作基础，完善公共服务，维护社会和谐稳定，着力营造人民群众安居乐业的良好环境。要做好政府工作，必须把强化服务作为第一目标，不断解放思想、转变观念、创新思路，敢于直面困难，善于破解难题，切实改进作风，努力提高执行力和公信力，树立廉洁奉公、求真务实的良好形象。

　　过去的五年，尽管我市经济社会发展取得了显著成就，但是我们也清醒地认识到，定西依然是全省乃至全国最贫困的地区之一，经济总量小、人均水平低、贫困比重大、基础条件差的基本市情还没有大的改变。经济社会发展中存在的突出困难和主要问题有：经济结构不合理，特色优势产业开发层次低，工业基础薄弱，现代服务业发展滞后，降能耗、减排放和保增长之间的矛盾更趋突出，优化经济结构、转变发展方式的任务十分艰巨。基础设施欠账大，生态环境还比较脆弱，事关长远发展的大项目、生产经营性项目较少，经济社会发展的内生动力不足。投资主体单一，融资平台建设滞后，财政收支矛盾突出。社会事业发展不平衡，事关人民群众切身利益的就业、医疗、教育、住房、社会管理等方面的问题有待进一步解决。政府职能转变不够到位，保障发展的体制机制不活，依法行政水平不高。一些部门和少数干部作风漂浮、效能低下，服务发展的大局意识、责任意识不强。对这些困难和问题，我们一定要采取得力措施认真加以解决。

　　今后五年发展目标和主要任务

　　今后五年，是定西争先进位的黄金期、蓄势转型的攻坚期、跨越发展的关键期。国家支持甘肃发展的政策效应加快释放，新一轮西部大开发战略深入实施，省委、省政府正在研究制定支持定西经济社会发展的意见，必将为我市加快发展注入新动力。经过多年努力，全市经济实力大幅提升，基础条件明显改善，为进一步加快发展奠定了坚实基础。刚刚闭幕的市第三次党代会提出了“立足新起点、实现新跨越，建设开放文明和谐新定西”的奋斗目标，绘制了今后五年发展的宏伟蓝图。我们一定要坚定信心，振奋精神，真抓实干，奋力开创经济社会发展新局面。

　　 政府工作的总体思路是：深入贯彻落实科学发展观，以转变经济发展方式为主线，坚持兴农强工、做大城镇、扩充总量、提升水平，精心打造“中国薯都”、“中国药都”，大力弘扬领导苦抓、部门苦帮、群众苦干的“三苦”精神，加快建设特色优势农畜产品生产加工、有色冶金、矿产建材、现代物流“四大基地”，全面建设宜居、特色、文化、活力、和谐新定西，努力加快农业产业化、工业化、城镇化、生态化和市场化进程。

　　经济社会发展的预期目标是：全市生产总值年均增长13%左右，到2016年达到340亿元，人均突破1万元。全社会固定资产投资年均增长30%以上，突破1000亿元，力争达到1500亿元。大口径财政收入年均增长20%以上，突破40亿元，力争达到45亿元。工业增加值年均增长20%以上，突破80亿元，力争达到100亿元。社会消费品零售总额年均增长17.5%以上，突破140亿元。城镇居民人均可支配收入年均增长11%以上，达到2万元。农民人均纯收入年均增长13%以上，达到5600元。城镇登记失业率控制在4%以内，人口自然增长率及万元生产总值能耗、主要污染物排放控制在省上下达的指标之内。

（一）着力统筹城乡发展，以改善基础条件支撑宜居定西建设。加快城镇、交通、水利、生态、新农村建设，不断改善城乡居民生产生活条件，推进城乡一体化发展。统筹市区、县城和重点小城镇建设，引导有条件的农村人口向城镇有序聚集，到2016年城镇化率提高到30%左右,建成区面积达到100平方公里以上。市区以打造园林城市、生态城市和地域文化特色城市为目标，加快推进旧城改造与新城建设步伐，力争完成投资200亿元以上。县城和重点小城镇实施一批供水、道路、垃圾处理、污水处理、园林绿化和集中供热等工程，努力增强综合承载功能，争取将临洮县纳入全国第三批小城镇发展改革试点县。完善交通运输体系，力争完成投资400亿元以上，开工建设宝兰客专过境段、陇西文峰至漳县大草滩2条铁路，临洮至渭源、渭源至武都2条高速公路和通渭至陇西、定西至郭城等15条二级公路，建成定临公路内官至临洮段、陇西文峰至漳县殪虎桥、徐合公路岷县过境段等3条二级公路，力争2016年国道全部实现高速化、省道和重要县道达到二级公路标准、县乡道达到四级油路标准、行政村基本通水泥路。加快建设天水至陇西、兰州至安定天然气管道支线工程，力争开工建设安定、陇西热电联产项目。引洮供水一期配套工程全面建成，二期配套工程启动实施。全面解决91万农村人口饮水安全问题，完成中小河流治理476公里，新增节水灌溉面积20万亩。加强渭河、洮河、漳河和关川河等流域生态治理，新修梯田150万亩以上，新增水土流失综合治理面积1980平方公里，治理程度达到50%，森林覆盖率达到16%，努力打造黄土高原水土流失综合治理示范区和全国生态文明示范区。积极探索扶贫开发与新农村建设相结合的有效机制，加强定点对口帮扶和社会扶贫工作，把农村低保制度与扶贫开发政策有效衔接起来，着力推进集中连片开发扶贫，争取沿洮扶贫开发示范带规划批复实施，易地搬迁贫困人口10万人以上，减少贫困人口20万人，把定西建成国家扶贫开发攻坚试验示范区。

　　（二）着力转变发展方式，以调整经济结构促进特色定西建设。全面推进质量振兴，提高经济发展质量和效益，逐步形成特色产业支撑、新兴工业引领、现代服务业跟进的地方经济体系，到2016年三次产业结构调整为21︰37︰42。立足打造 “中国薯都”，着力抓好种薯繁育、精深加工、产品研发等关键环节，把定西建成全国马铃薯贸工农一体化示范区，把西川农业科技园区建成国家级马铃薯良种生产示范园区。立足打造“中国药都”，促进中医药产业由原料生产、初级加工向标准化种植、无公害生产、园区化加工转变，把定西建成全国重要的中药材药源基地、中医药加工和现代制药基地、中药材现代物流集散地、大宗中药材价格形成中心和现期货交易市场，力争到2016年马铃薯、中医药产业产值分别突破50亿元和100亿元，争取把马铃薯、中医药循环经济产业园升格为国家级园区。大力发展旱作高效节水农业，加快科技成果转化，进一步加大全膜双垄沟播技术推广力度，稳定提高粮食产量。紧密结合牛羊养殖大县建设，加快草畜转化步伐，着力发展草食畜牧业。调整优化规模养殖布局，完善鼓励扶持政策，推动畜禽养殖与玉米秸秆高效利用、肉食品加工一体化发展，力争规模养殖比重提高到60%以上，努力把定西建设成“兰白都市经济圈”绿色无公害肉菜蛋奶供应基地。因地制宜培育发展区域性支柱产业，加快形成“一乡一业”、“一村一品”发展格局。按照依法、自愿、有偿原则，积极稳妥推进土地流转，促进设施农牧业、特色优势产业和区域性支柱产业步入规模经营，不断提升农业比较效益。充分发挥工业在转变经济发展方式中的引领作用，全力实施工业发展“十大行动计划”， 加快壮大铝冶炼及加工、中医药、马铃薯精深加工、矿产建材、盐化工、电能及新能源、装备制造等七大主导产业，确保工业增加值年均增长20%以上、力争达到30%，占生产总值的比重和对财政的贡献率均提高到30%以上。加快推进开发区和工业集中区建设，力争到2016年建成销售收入超100亿元园区1个、超50亿元园区3个。突出产业及园区招商，高度重视招商项目落地工作，力争招商到位资金年均增长35%。认真落实扶持非公有制经济发展的政策措施，拓宽市场准入领域，完善管理服务体系，大力培育投资主体，实施全民创业工程，积极解决土地、资金、人才等突出问题，新发展民营企业1800户，培育上市公司3户以上，力争到2016年非公有制经济增加值占生产总值的比重达到45%。实施 “十园百市千店”工程，规划建设一批区域性物流中心，突出抓好定西现代物流园区和甘肃定西国家级马铃薯专业市场、陇西宇臻物流公司市场改扩建等重点项目建设，积极引进和培育大型物流集团，不断完善城乡现代流通服务体系，力争到2016年物流业增加值占生产总值的比重达到10%以上。

　　（三）着力打造特色文化大市，以提升文明水平引领文化定西建设。深化文化体制改革，大力发展文化事业，培育新兴文化产业，促进文化资源优势转化为产业优势，努力打造陇中特色文化大市。大力实施文艺精品战略，鼓励文艺工作者面向发展实践创作富有地域特色的文艺精品。加强传统文化发掘保护与开发管理，建立健全非物质文化遗产抢救、保护机制。加快文化馆、图书馆、博物馆、美术馆等公共文化服务设施建设，规划建设临洮马家窑彩陶文化、陇西李氏文化、岷县洮砚文化及通渭书画产业园区，打造产业聚集发展平台。培养和造就一批文化经纪人和民间工艺大师、优秀工匠，支持彩陶、洮砚、剪纸等民间工艺品生产加工。加强对传统优势产业的数字化、信息化改造，大力发展现代传媒、演艺娱乐、网络文化、会展服务等新兴文化产业，力争文化产业增加值年均增长20%。充分挖掘特色文化内涵，促进文化与旅游深度融合，着力打造旅游品牌。积极申报贵清山/遮阳山国家地质公园、国家级风景名胜区，争创国家5A级旅游景区。联手构建定西、兰州、天水一体化运作机制，加快发展自然风光游、革命胜迹游、人文古迹游、陇中民俗风情游和生态休闲度假游，力争旅游接待人数和旅游收入年均增长20%以上，努力把定西打造成“兰白都市经济圈”的重要休闲度假基地。

　　（四）着力优化发展环境，以创新体制机制助推活力定西建设。加大财税金融、社会事业等重点领域改革，着力消除各种体制机制障碍，切实增强跨越发展的内在活力和动力。继续推进财政体制改革，整合地方财力，优化支出结构，集中力量办大事。努力破解融资难题，创新投融资体制，进一步健全完善政府信用体系，探索林权、土地承包经营权、宅基地使用权、特许经营权、商标专用权抵质押等新型贷款方式，探索组建投资集团，搭建投融资平台，多渠道增加城市建设、产业开发、工业发展等方面资金投入。完善金融服务体系，争取证券、信托、基金等金融机构在我市设立分支机构，不断壮大金融业实力。积极探索启动民间资本的有效措施，引导民间资本投向经济社会发展重点领域和薄弱环节。健全土地、资本、劳动力、技术、信息等要素市场，推进多层次市场体系建设。加快科技创新和成果转化应用步伐，充分发挥科技对经济发展的引领和支撑作用。正确处理发展经济与保护环境的关系，全面推行安全、清洁和节约生产，高度重视水、土地、矿产、林草等资源的保护和集约节约利用，大力发展循环经济，下决心降低能耗、减少排放，提高生态文明建设水平。下沉工作重心，前移管理关口，改进服务方式，推动社会管理在创新和加强中不断取得实效。

　　（五）着力增进群众福祉，以完善公共服务保障和谐定西建设。高度重视物价调控工作，建立价格调节基金，认真落实保供给、畅流通、强监管等各项措施，探索建立平抑价格长效机制，确保全市物价水平基本稳定。全面落实各项惠民政策，促进城乡居民收入持续增长。千方百计扩大就业，大力扶持城乡劳动力创业，年输转劳动力60万人次。不断完善城乡社会保障体系，城乡低保实现应保尽保，社会基本养老保险实现全覆盖。严格执行优抚安置和社会救助政策，新建福利机构及老年活动中心200个，五保集中供养率提高到40%以上。调整优化中小学校布局，加强寄宿制和标准化学校建设，基本解决20万寄宿生的食宿问题。加快普及高中阶段教育，促进高等教育、职业教育、城乡教育持续发展，支持民办教育和特殊教育，重视和加强学前教育。大力开展全民健身活动，促进群众体育和竞技体育协调发展。深化医药卫生体制改革，完善新型农村合作医疗、城镇居民基本医疗和大病医疗救助制度，加快医疗机构基础设施建设，有效解决群众看病难看病贵问题。加强人口和计划生育工作，稳定低生育水平。健全住房保障体系，新建各类保障性住房1.96万套、127万平方米，改造农村危旧房9.6万户。进一步加强防灾减灾救灾工作，妥善安排受灾群众的生产生活。实施妇女阳光家园工程，建设农村留守儿童之家，努力发展残疾人和老年人事业。

　　2012年政府工作建议

　　2012年是新一届政府任期的第一年。政府工作要以市第三次党代会精神为统领，全面组织实施“十二五”规划纲要，一心一意谋发展，凝心聚力抓落实，努力为经济社会实现新跨越奠定基础。

　　经济社会发展的预期目标是：生产总值增长13%左右，固定资产投资增长30%，大口径财政收入增长20%，工业增加值增长23%，社会消费品零售总额增长17.5%，城镇居民人均可支配收入增长11%，农民人均纯收入增长13%，人口自然增长率控制在7‰以内，居民消费价格涨幅控制在5.5%左右，城镇登记失业率控制在4%以内，节能减排指标控制在省上下达的目标之内。

　　围绕上述目标，重点抓好以下工作：

　　一是突出抓好项目建设。着力加强项目衔接争取，力争514个重点项目列入省投资计划，争取国家政策性资金60亿元以上，完成固定资产投资368亿元，力争实施500万元以上项目1000个。积极筹措前期费5000万元，力争新储备项目1000个以上，其中50%以上完成可研编制，不断充实项目储备库。配合抓好省部属重大项目建设，全面完成引洮供水一期及配套工程建设任务，确保引洮供水一期工程年底前建成通水，编制完成引洮工程水资源综合利用规划，启动实施市区东河、西河防洪工程建设。进一步加强和改进项目管理，开展项目建设质量年活动，严格落实各项法规政策和工作制度，确保各类项目建设在保证实施质量和资金安全的前提下，加快建设进度，尽早发挥效益。继续办好马铃薯、中医药产业发展大会，坚持走出去与引进来相结合，依托兰洽会、西洽会等省内外重点节会，全力抓好招商引资工作，不断创新招商引资方式，切实提高项目履约率，力争引进到位资金115亿元以上，增长35%。

　　二是大力发展现代农业。继续巩固农业基础地位，高度重视粮食生产，稳定农作物播种面积。坚持鲜薯生产与良种生产并重、重点突破良种生产，销售鲜薯与加工并重、重点突破高端产品研发，千家万户种植与企业示范推广种植并重、重点突破企业跟进生产，规模种植与提高质量并重、重点突破均衡上市和多元发展，不断提高马铃薯产业开发水平。完成西川国家级马铃薯良种示范园区实施规划，理顺管理体制。大力推行标准化生产，建立马铃薯一级种扩繁基地22万亩、原种扩繁基地 1.8万亩，中药材标准化基地50万亩、优质种子种苗基地4.2万亩。稳定中药材种植面积，提高标准化种植比例，依托园区提升中医药产业发展规模和水平。采取更加有力的措施，狠抓草畜转化工作，加快发展规模养殖业，新建标准化养殖小区85个、规模养殖企业65个、规模养殖户8000户，完成玉米秸秆青贮氨化150万吨，努力提高畜牧业在农业内部的比重。结合引洮工程通水后水资源的科学利用，编制完成高效节水农业规划，大力发展旱作农业和设施农业，完成全膜双垄沟播322万亩，新建日光温室1000座、塑料大棚2000亩。积极推广测土配方等重点适用技术，建设优质农产品生产加工基地，培育专业合作经济组织，进一步提升农业产业化经营水平。

　　三是加快发展地方工业。进一步加大园区建设投入力度，完成固定资产投资36亿元，其中基础设施建设投资5.6亿元，新引进项目30个以上。市循环经济园区完成起步区道路、桥梁及河道治理、土地整理等项目，启动建设城东一路、城北五路，争取新引进企业5户以上，组建运营房产开发公司和融资担保公司。推进马铃薯循环经济产业园建设，确保爱兰公司3万吨马铃薯速冻薯制品、蓝天公司变性淀粉及壁纸胶、金大地二期薯条生产线等项目建成投产。加快陇西中医药产业园建设，力争千金药业、丽珠参源、奇正藏药等企业建成投产，园区产值突破5亿元。坚持技改、续建、新上并举，实施1000万元以上工业项目75个，完成投资53亿元，确保华能华家岭风电一期等28个项目建成投产，祁连山集团二期180万吨新型干法水泥等47个项目完成年度计划，康美药业公司50万吨中药材仓储现代物流园及加工等12个项目开工建设。扶持重点企业发展，加快漳县食用盐国家储备库报批和华腾石油机械公司战略重组步伐。大力实施名牌战略，争创甘肃省名牌产品5个。认真落实预警监控措施，全面完成节能减排目标任务。

四是努力提升第三产业。实施好狼渡滩湿地草原、李家龙宫等50万人次景区创建工程，进一步开发洮河漂流、温泉洗浴、中医药养生、农家乐等旅游项目，提升节会旅游和乡村休闲旅游档次，力争接待游客320万人次，旅游综合收入达到12亿元。充分发挥交通区位优势，引进和培育大型物流企业2户以上，启动建设西部汽车商贸城项目，促进现代物流业加快发展。实施“万村千乡市场工程”，抓好3个农产品交易市场升级改造，建设标准化农家店150户，着力构建新型农村流通网络和配送体系。加大房地产业调控引导力度，加强商品房预售管理，促进房地产市场平稳健康发展。大力发展家政服务、文化娱乐、邮政通信等便民服务业，不断完善社区服务体系。

　　五是统筹推进城乡建设。市区争取实施各类建设项目85个，完成投资50亿元以上。抓好定西公园、文化艺术中心、博览中心、游泳馆和14个机关事业单位搬迁工程建设。加快气象新村等“城中村”改造，配套完善旧城区供热、供水、环卫等基础设施，加大绿化美化亮化力度，建立河道治理、交通秩序、市容市貌综合整治长效机制。各县县城和重点小城镇实施建设项目173个，完成投资50亿元。加强农村基础设施建设，新修梯田30万亩，新增节水灌溉5万亩、水土流失综合治理面积350平方公里，完成病险水库除险加固4座，实施林业重点工程20万亩、面山绿化4万亩，继续推进平定、天定高速公路生态长廊建设。大力推广新能源，新建农村户用沼气4800户。加强农村环境整治，实施连片整治项目44个。抓好14 个易地扶贫搬迁项目、2个省级连片开发试点和60个整村推进项目，稳步推进新农村建设。

　　六是注重强化财金保障。积极培育财源，加强税收征管，确保财政收入稳步增长。争取专项资金和财力补助，不断优化财政支出结构。采取财政贴息、补助、担保、奖励等措施，引导更多金融信贷、社会资本、民间投资向经济社会发展重点领域集聚，实施亚行贷款市区道路等5个国外贷款项目。加强融资平台建设，支持各类担保公司做大做强，争取发展各类小额贷款公司10家以上，进一步规范民间借贷，提高融资水平。积极开展“引行入定”工作，争取兰州银行在陇西、岷县设立支行，浙江民泰银行在我市挂牌运营。加大扶持、引导和监管力度，促进商业保险健康发展。全面启动“农村信用信息管理系统”，实施信用惠农工程，优化农村金融环境。

　　七是全面发展社会事业。推进学校布局结构调整，改善办学条件，实施义务教育阶段学校改扩建、学前教育、教师周转宿舍、职业教育基础能力建设等项目，下决心加快市区普通高中和幼儿园建设，着力解决入学入园难的问题。加快科技创新步伐，强化产学研结合，推动科技成果转化应用。深化医药卫生体制改革，推进国家基本药物招标采购和统一配送，抓好公立医院改革试点，加快城乡居民健康档案信息化建设步伐，建成市医院迁建项目，改扩建2所妇幼保健院综合楼。深化文化体制改革，建成市级文化馆、图书馆、博物馆和剧院，培育文化产业龙头企业10户以上，文化产业增加值增长20%以上。积极发展广播电影电视事业，新增有线电视数字化整体转换2.5万户。大力发展老年人、青少年、妇女儿童和残疾人事业。

　　八是切实加强社会管理。进一步加强信访工作，畅通群众诉求表达渠道，深入排查化解各类社会矛盾，建立重大工程项目建设和重大政策制定风险评估制度，健全公共和社会安全事件预防预警机制，提高突发事件应急处置能力。深入开展普法教育，切实提高公民法律意识。全面落实企业安全生产主体责任，有效预防重特大事故发生。整顿规范市场秩序，强化食品药品监管，严厉打击价格违法行为，确保食品药品质量安全和物价稳定。加强城市基础设施管理维护，探索建立农村道路、小型水利等公益性项目有效管理方式。引导规范和发展各类社会组织，充分调动人民群众参与社会管理的积极性。广泛开展双拥共建活动，加强军政军民团结。加强社会治安综合治理，依法严厉打击各类违法犯罪，提高人民群众的安全感和满意度。

　　九是竭力为民办好实事。本着量力而行、尽力而为、注重实效的原则，在广泛征求意见的基础上，2012年继续为广大人民群众办好十件实事。（1）实施校安工程16.5万平方米。（2）在市区新城区建设1所高级中学和1所幼儿园。（3）建成5所县级卫生监督机构，为基层医疗机构配置新型医疗设备，培训基层医疗机构全科医生100名，免费为全市20万育龄妇女开展生殖健康服务。（4）新建农家书屋313个。（5）建成通村水泥路500公里，全市行政村通水泥路或沥青路率达到45%。（6）实施农村安全饮水工程,解决20万人的饮水安全问题。（7）发放租赁补贴14943户，建设保障性住房4600套，改造农村危旧房1.8万户。（8）在全市40个乡镇安装视频监控系统，为困难群众办理法律援助案件350件。（9）新增小额担保贷款2亿元，支持创业4000人，带动就业8000人以上；重点扶持100名择业期满未就业的城乡特困家庭高校毕业生就业。（10）在市区15个小区设立30个车载蔬菜进社区直销店。　　全面加强政府自身建设推动定西经济社会跨越发展，对政府工作提出了新的更高的要求。我们一定要着力转变政府职能，理顺职责关系，优化组织结构，完善管理体制，全面加强自身建设，不断提高行政能力和服务水平。

　　（一）坚持解放思想，强化学习培训。倡导学以致用的良好风气，培养终身学习的意识和自觉性。采取走出去、请进来方式，有针对性地加大干部学习培训力度，重点举办经济常识、项目管理、工业发展、招商引资、城镇建设、行政执法等培训班，切实提高各级干部驾驭经济、推动发展的能力和水平。注重加强对法律法规、各项政策、资本运作、现代管理和外地经验的学习，使思想观念、思维方式和工作方法适应加快发展的要求。

　　（二）坚持执政为民，转变政府职能。着力强化政府社会管理和公共服务职能，加快推进政企分开、政事分开、政资分开、政府与市场中介组织分开，以优质服务吸纳和聚集生产要素，推动经济社会快速发展。加快行政审批制度改革，大力推行阳光政务，加强政府信息化建设和政务公开，实行审计结果公告制，适时公开国民经济计划和财政预算执行情况，畅通信息公开渠道，不断完善便民服务体系。

　　（三）坚持依法行政，规范行政行为。自觉接受人大、政协和社会各界监督，提高人大代表建议、政协委员提案办理质量。完善政府行政管理决策机制，健全重大事项专家论证、技术咨询、决策评估和公示听证制度，推进行政管理决策科学化、民主化。进一步加强政府法制建设，全面推进行政执法责任制，加大行政执法监督力度，加强行政监察和审计监督，提高各级政府依法行政、依法办事能力。

　　（四）坚持真抓实干，提高行政效能。大力弘扬求真务实和雷厉风行的工作作风，着力解决懒散拖拉、推诿扯皮等问题，把每件实事、每个项目和每项工作抓紧抓实、抓出成效。建立健全绩效管理、考核评估、督查督办、行政问责制度，严肃查处乱摊派、乱收费和吃拿卡要等影响发展环境的人和事，形成奖优罚劣、跟踪落实、失责必问的行政责任追究机制和工作推进机制，确保各项工作有效落实。

　　（五）坚持廉洁从政，树立良好形象。全面落实党风廉政建设责任制，深入推进惩治和预防腐败体系建设。加强干部教育监督管理，严格遵守廉洁自律各项规定，做到清白做人、干净做事。树立过紧日子思想，严格控制会议、用车、接待等经费，降低行政成本，勤俭办一切事情。切实加强对工程建设招投标、强农惠农政策执行、社会保障资金使用、政府采购等情况的监督检查，确保阳光操作、规范管理、安全运行。

　　各位代表，难得的机遇和人民的期待召唤着我们，神圣的职责和崇高的使命激励着我们。让我们在省委、省政府和市委的坚强领导下，在市人大和市政协的监督支持下，以更加振奋的精神、更加务实的作风、更加有力的举措，锐意进取，扎实工作，为建设开放文明和谐的新定西而努力奋斗！
